# Heavy Foil

**Published:** 1870-03

**Authors:** 


### HEAVY FOIL.

We have been using heavy foil constantly for the last month, and somewhat for the last nine months.

We began with 10, after some experiments with this took up 20, then 30, then 60, and finally 120. Of these we now use daily 30, 60 and 120, and No. 4 for light foil.

With the present experience, we can hardly realize how we ever got along without heavy foil; indeed, we did not get along anything like so satisfactorily as now.

Our first impressions about heavy foils were very unfavorable, but we have long ago learned not to regard first impressions as the ultimatum, our first impressions are almost always based upon partial or fragmentary testimony,and every thinker will understand how easy it is to be mistaken by knowing only part of the truth, and how often our opinions have to be changed and modified by the successive presentation of new facts, truths and testimony.

The man who boasts that he never changes his opinions or views, gives evidence that he is not capable of receiving or understanding any additional truth or testimony, or that he is in possession of all there is upon the subject in question, which is a pretty high position for any one to take.

Now, in regard to heavy foi's, our first impressions were that they would be difficult to work, difficult to weld, and not readily adapted to the walls of the cavities to be filled ; but the truth developed by experience subverted all these impressions.

The heavy foil welds better than the light, is not so readily cut up and comminuted, but is tough and adherent, and welds very perfectly. Its adaptation, we think, is as good, if not better, than the thin. The method of manipulation, of course, is different, and we hardly feel like incurring the risk of leading our brethren astray by attempting to describe the precise method of manipulation required. We think the better plan is (for every one who wishes to learn the precise mode of using heavy foils), to witness the operation, and in that way attain the right method at once.

We have no hesitation in saying that to correctly obtain this one item of practice is amply worth the expense and labor of a journey of a thousand miles.

The difference in the appearance of our fillings of to-day and those of six months ago, is very marked indeed, and chiefly owing to the use of heavy foils. If hereafter we shall feel that our description of the manner of using heavy foil would do more good than *evil*, we may make the attempt; but for the present, *quantum sufficit.* T.

